# Changes in the Flower and Leaf Proteome of Common Buckwheat (*Fagopyrum esculentum* Moench) under High Temperature

**DOI:** 10.3390/ijms22052678

**Published:** 2021-03-06

**Authors:** Przemysław Kopeć, Marta Hornyák, Jakub Pastuszak, Anna Szczerba, Marcin Rapacz, Jacek Waga, Agnieszka Płażek

**Affiliations:** 1 Polish Academy of Sciences, Institute of Plant Physiology, Niezapominajek 21, 30-239 Kraków, Poland; p.kopec@ifr-pan.edu.pl; 2 Department of Plant Breeding, Physiology and Seed Science, University of Agriculture, Podłużna 3, 30-239 Kraków, Poland; marta.golebiewska@gmail.com (M.H.); jakub.pastuszak@urk.edu.pl (J.P.); anna.szczerba21@gmail.com (A.S.); j.waga@urk.edu.pl (J.W.); rrplazek@cyf-kr.edu.pl (A.P.)

**Keywords:** common buckwheat, high temperature, proteomics, heat-affected proteins

## Abstract

Common buckwheat (*Fagopyrum esculentum* Moench), a pseudocereal crop, produces a large number of flowers, but this does not guarantee high seed yields. This species demonstrates strong abortion of flowers and embryos. High temperatures during the generative growth phase result in an increase in the degeneration of embryo sacs. The aim of this study was to investigate proteomic changes in flowers and leaves of two common buckwheat accessions with different degrees of heat tolerance, Panda and PA15. Two-dimensional gel electrophoresis and mass spectrometry techniques were used to analyze the proteome profiles. Analyses were conducted for flower buds, open flowers capable of fertilization, and wilted flowers, as well as donor leaves, i.e., those growing closest to the inflorescences. High temperature up-regulated the expression of 182 proteins. The proteomic response to heat stress differed between the accessions and among their organs. In the Panda accession, we observed a change in abundance of 17, 13, 28, and 11 proteins, in buds, open and wilted flowers, and leaves, respectively. However, in the PA15 accession there were 34, 21, 63, and 21 such proteins, respectively. Fifteen heat-affected proteins were common to both accessions. The indole-3-glycerol phosphate synthase chloroplastic-like isoform X2 accumulated in the open flowers of the heat-sensitive cultivar Panda in response to high temperature, and may be a candidate protein as a marker of heat sensitivity in buckwheat plants.

## 1. Introduction

Common buckwheat (*Fagopyrum esculentum* Moench), which belongs to the *Polygonaceae* family, is a valuable source of rutin, iron, dietary fiber, and many other microelements. Buckwheat seeds do not contain gluten and have a well-balanced amino acid composition rich in lysine [1,2]. This species does not require good soil; however, it is sensitive to a number of environmental factors, such as frost and cold, high temperature, dry wind, and drought [3]. Its flowering biology is complex, as it is a heterostylic species that produces pin and thrum flowers. The flowers need to be cross-pollinated by insects, mainly bees. This plant is characterized by strong self-incompatibility. A single flower is able to be fertilized for one day only [4]. Buckwheat blooms throughout the growth season, but its abundant flower production (up to 2000 flowers per plant) does not guarantee high seed yields [2,5,6]. Our previous research showed that buckwheat plants have a limited ability to fill seeds, and hence this species shows a strong abortion of flowers and embryos [7].

In earlier investigations, we found that buckwheat plants at the vegetative phase develop much better at a higher temperature (30 °C) than at 20 °C [8]. However, 30 °C is too warm for optimal embryo development [9]. In our previous work [9], we detected a significantly higher degree of embryo sac degeneration in plants grown at 30 °C than in plants grown at 20 °C.

High temperature negatively affects metabolic processes, protein structure, electron transport in cytoplasmic membranes, and the energy status of photosystems, while inducing the formation of reactive oxygen species (ROS) [10]. In addition, heat stress is associated with an enhanced risk of improper protein folding and denaturation of several intracellular and membrane proteins [11]. Heat leads to the increased expression of several proteins, especially those in the large heat-shock protein (HSP) family, which includes high molecular mass HSPs (from 6 to 110 kDa) and small HSPs (from 15 to 45 kDa) [11,12]. Proteomic analyses have shown that many other proteins are also synthesized in plants during adaptation to high temperature; examples include proteins involved in the antioxidant system [13,14,15], enzymes involved in biosynthesis of UDP-glucose, pyruvate dehydrogenase, transketolases, and enzymes in the Krebs cycle and pentose phosphate pathway that regenerate ribulose-1,5-bisphosphate (RuBP) and activate Rubisco [12].

Previous studies have focused on changes in proteomes during embryogenesis, fertilization, and seed formation [16]. Feng et al. [17] analyzed the proteome of *Arabidopsis thaliana* flowers, and proteins involved in protein synthesis, folding, modification and degradation, as well as a belonging to the regulatory system. Kerim et al. [18] analyzed the proteome of rice at several male gametophyte stages: the pollen mother cell tetrad, early young microspores, the early and late binucleate stages, and the heading stage. The proteins they observed, which played an important role in the development of the male gametophyte, were related to the metabolism of sugars, cell elongation and cell expansion. Although it is more difficult to analyze proteins in the female gametophyte because of technical difficulties, Uchiumi et al. [19] detected some proteins in rice eggs: the cytoplasmic glycolytic enzyme glyceraldehyde-3-phosphate dehydrogenase, histone H4, cytoplasmic ascorbate peroxidase, and a member of the HSP 90 subfamily. Feng et al. [17] detected differences in the abundance of pistil-proteins between compatible and self-incompatible *Prunus armeniaca* cultivars. Liu et al. [20] identified more than 40 proteins in aborted seeds, including three cysteine proteases that were possibly involved in programmed cell death. Das et al. [21] detected the differential expression of 44 abiotic stress-responsive proteins in soybean leaves under several abiotic stresses. The results and observations of those studies suggest that many differentially expressed photosynthesis-related proteins disrupt the regulation of Rubisco, electron transport, and the Calvin cycle during exposure to abiotic stresses.

The aim of this study was to investigate proteomic changes in flowers and leaves of two common buckwheat accessions, the Panda cultivar and the PA15 breeding line, which have different degrees of heat tolerance. In our previous study [9], we showed that Panda is more sensitive to heat stress than PA15. Our results showed that there were more degenerated embryo sacs at the flower bud formation stage at 30 °C than at 20 °C in Panda. By contrast, in PA15, the number of degenerated embryo sacs only increased after a longer duration of heat stress, i.e., at the open flower phase. In this study, we explored the proteomic changes during flower development, and the differences in proteomes between high temperature (30 °C) and control (20 °C) conditions. In our study, we mainly wanted to compare the stress-induced changes in the proteome in two accessions with different degrees of tolerance. On this occasion, we wanted to further identify heat-related proteins. To study the proteome we used two-dimensional electrophoresis in combination with liquid chromatography-mass spectrometry (nanoLC-MS/MS) and peptide mass fingerprinting (PMF). Although there have been advances in the methodology of proteome research in recent years, the methods used in our study are still adequate and widely used in studies similar to ours [22,23,24,25]. We identified proteins showing differences in abundance in response to heat in the two buckwheat accessions. Our results shed light on the mechanisms responsible for tolerance to heat stress, as manifested by a lower degree of degraded embryo sacs under high temperature. These analyses were performed for flower buds, open flowers capable of fertilization, and wilted flowers, as well as donor leaves, i.e., those growing closest to the inflorescences.

## 2. Results

### 2.1. Protein Profiles in Flowers and Leaves

**Figure 1:**
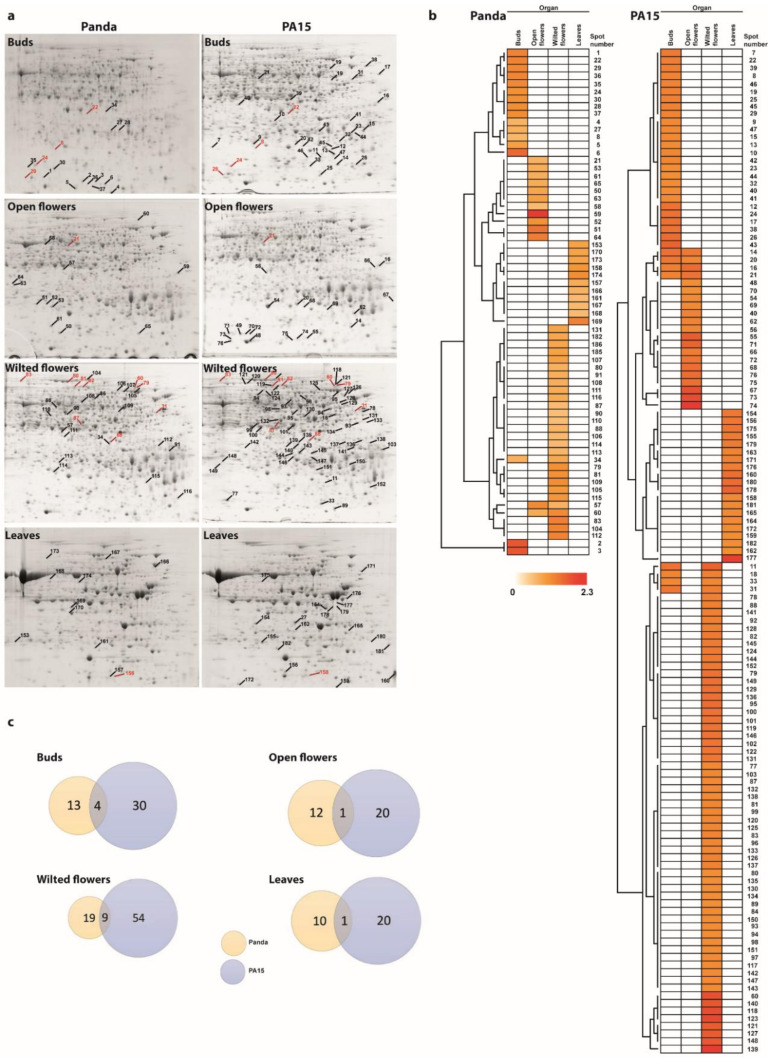
Changes induced by high temperature in proteomes of flower buds, open flowers, wilted flowers, and donor leaves of two common buckwheat accessions, Panda and PA15: (**a**) Representative two-dimensional gel electrophoresis (2-DE) gels of total proteins; proteins were separated by isoelectric focusing on an immobilized pH gradient IPG strip (pH 4–7) followed by sodium dodecyl sulfate (SDS)-PAGE on a 12% acrylamide gel; marked spots are those showing changes in abundance in heat-stressed plants compared with control plants, red color indicate additionally proteins common for both Panda and PA15; (**b**) heatmaps illustrating the results of hierarchical clustering analysis using Euclidean and Ward’s linkage methods; cluster analysis was conducted using PermutMatrix software v.1.9.3. (http://www.atgc-montpellier.fr/permutmatrix/); colors correspond to log-transformed values of protein spot fold-change; (**c**) Venn diagrams comparing proteome profiles (up-regulated proteins only) between two buckwheat accessions.

In the 2-D electrophoresis analyses, different numbers of proteins were identified depending on the plant organ and the accession. Representative 2-D protein patterns are shown in Figure 1a. In the 2-D maps of flower bud proteins, we detected 1189 protein spots in Panda and 1159 in PA15. There were fewer protein spots in the 2-D maps produced from open flowers, namely 900 for Panda and 977 for PA15. In the 2-D maps produced from unpollinated wilted flowers, there were 1117 protein spots for Panda and 1097 for PA15. The smallest numbers of protein spots were detected in donor leaf samples, namely only 701 for Panda and 826 for PA15.

High temperature did not affect the number of protein spots within the accessions, but it affected the abundance of some proteins. In flower buds, high temperature up-regulated the expression of 17 proteins in Panda and 34 in PA15. Fewer proteins were up-regulated by heat in the open flowers: 13 in Panda and 21 in PA15. High temperature strongly affected the proteome of wilted flowers, up-regulating the expression of 28 proteins in Panda and 63 in PA15. In the donor leaves, heat stress affected the expression of 11 proteins in Panda and 20 in PA15. On the other hand, no downregulation of the expression of specific proteins as a result of high temperature was observed in any case.

The proteomic response to heat stress differed among plant organs and between the two accessions, as illustrated by heat maps and the results of hierarchical clustering analysis using Euclidean and Ward’s linkage methods (Figure 1b). In Panda, only three proteins were commonly up-regulated by heat stress in different plant organs (in two out of four organs only). In PA15, eight proteins were commonly up-regulated by heat stress in two organs. A few proteins were commonly up-regulated in Panda and PA15 by heat stress, but most of the proteins up-regulated by heat stress differed between the two accessions. Venn diagrams illustrating these results are shown in Figure 1c. We detected four proteins up-regulated in buds of both accessions, one in open flowers, nine in wilted flowers, and one in donor leaves. In each accession, no proteins showed heat-inducible expression at all stages of flower development. In Panda, the abundance of spot 34 changed in the flower buds and wilted flowers in response to heat stress, and spots 57 and 60 were up-regulated by heat stress in open flowers and wilted flowers. In PA15, five protein spots (no. 14, 16, 18, 20, and 21) showed changes in abundance under heat stress in flower buds and open flowers, and three spots (no. 11, 31, and 33) showed changes in abundance under heat stress in flower buds and wilted flowers.

### 2.2. Identification of Differentially Accumulated Proteins

To qualitatively analyze the protein spots on the two-dimensional gel electrophoresis (2-DE) gels, 182 spots were excised from the gels and analyzed using nanoLC-MS/MS. Only 31 proteins were successfully identified and annotated with the functions of homologous proteins. Next, PMF analysis of protein spots that were not identified by nanoLC-MS/MS identified another 42 proteins. The results are listed in Table 1. For the remaining unidentified spots, searches were performed against the Swiss-Prot database, searching for proteins among all taxa. Based on the identified proteins, we searched for homologous proteins among green plants using the BLASTP program. This procedure allowed us to identify seven additional plant proteins (Table 2).

**Figure 2:**
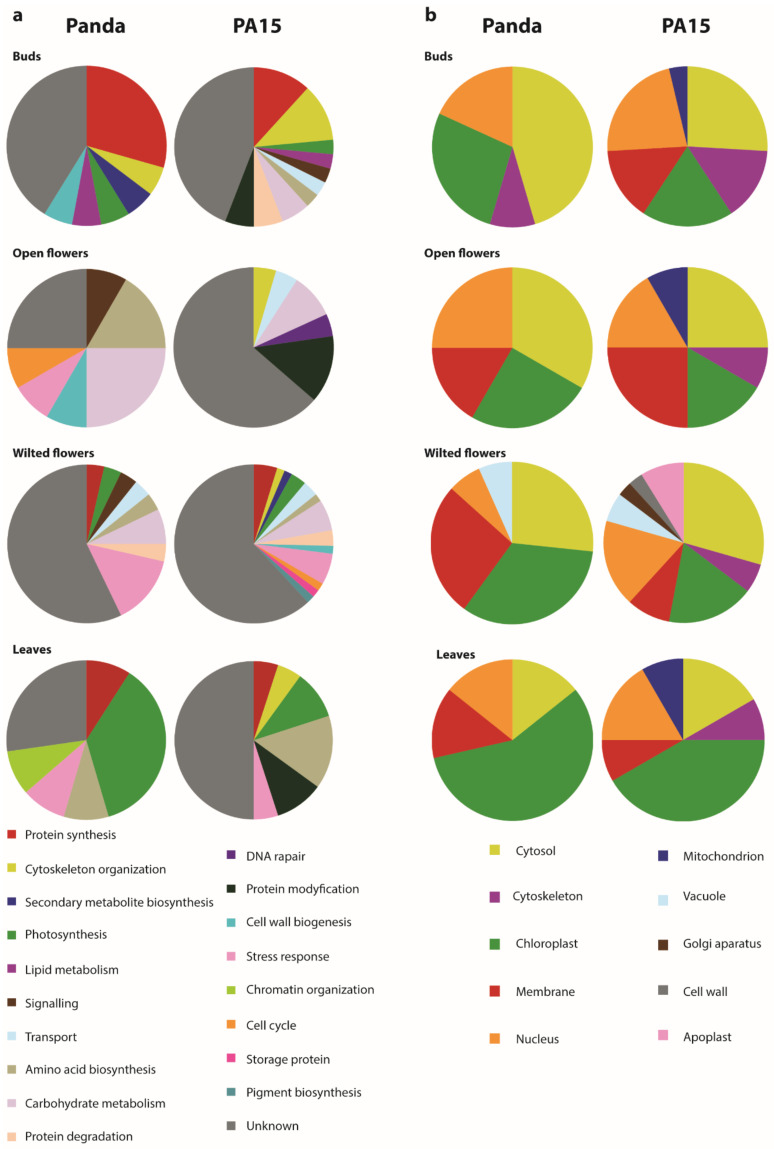
Composite graph showing heat-affected proteins in different organs of Panda and PA15 buckwheat accessions: (**a**) Biological functional categorization of heat-affected proteins; (**b**) distribution of identified proteins according to subcellular location; proteins with two and more localizations in the cell were assigned to all places. Biological function and subcellular localization were assigned based on information in the UniProt database.

Using the information from the UniProt database, we assigned the biological function (Figure 2a) and subcellular location (Figure 2b) to the identified proteins. Some proteins were assigned to more than one subcellular location, which was reflected in the plots.

In flower buds, heat stress mainly caused an increase in the abundance of cytosol-localized proteins related to protein synthesis. In Panda, these were mainly ribosomal proteins, whereas in PA15, two out of four proteins were transcription factors. Other heat-affected proteins in PA15 were related to organization of the cytoskeleton. Three proteins were up-regulated by heat stress in both Panda and PA15: two proteins involved in protein synthesis (60S ribosomal protein L5-1 and trihelix transcription factor GTL2) and one involved in lipid metabolism (acyl-[acyl-carrier-protein] desaturase 6).

For open flowers, the majority of the proteins showing changes in abundance under heat stress were related to carbohydrate metabolism in Panda, and protein modification in PA15 (Figure 2a). In Panda, most of the heat-affected proteins were localized to the cytosol, but some were localized to the chloroplast, plasma membrane, and nucleus (Figure 2b). In PA15, more of the heat-affected proteins were localized to the plasma membrane than to the cytosol, cytoskeleton, chloroplasts, mitochondria, and nucleus. Only one protein in open flowers, 6-phosphogluconate dehydrogenase, was up-regulated by heat stress in both buckwheat accessions. This enzyme is involved in the pentose phosphate pathway. Among all of the heat-affected proteins, indole-3-glycerol phosphate synthase showed the largest increase in abundance under heat stress. In Panda, its abundance in open flowers of plants grown at 30 °C was 213-fold that in plants grown at 20 °C. This enzyme is involved in the biosynthesis of the precursor of indole ring-containing compounds. It was identified using the BLASTP program on the basis of its homology to indole-3-glycerol phosphate synthase in *Acidithiobacillus ferrooxidans.*

Only a few of the heat-affected proteins in wilted flowers were identified. The identified proteins were mainly involved in the stress response (Figure 2a). Two 70 kDa heat-shock proteins and the glutamate receptor 3.4 were up-regulated by heat stress in wilted flowers of both buckwheat accessions. The other common proteins were chaperone protein ClpC, V-type proton ATPase catalytic subunit A, calpain-type cysteine protease DEK1, phosphoglycerate kinase, and fructose-bisphosphate aldolase 2. In PA15, several proteins related to carbohydrate metabolism were up-regulated under heat stress. The identified heat-affected proteins in wilted flowers of Panda were mainly localized in chloroplasts, while those in PA15 were mainly localized in the cytosol (Figure 2b).

In the donor leaves, high temperature up-regulated proteins related to photosynthesis in Panda (Figure 2a). Most of the identified heat-affected proteins in the donor leaves of Panda and PA15 were localized in the chloroplast (Figure 2b). Many of the heat-affected proteins in the donor leaves of PA15 were not identified, but most of the identified proteins were involved in amino acid biosynthesis. The cytosol-localized enzyme S-adenosylmethionine synthase showed changes in abundance in donor leaves in both accessions under heat stress.

All results obtained in this experiment are summarized in detail in Table S1.

## 3. Discussion

A central role in plant thermotolerance is played by reactive oxygen scavenging enzymes, heat-shock proteins (HSPs), and heat stress-responsive transcription factors (HSFs), which induce expression of HSPs, signal and regulatory proteins, proteins involved in metabolism, and redox homeostasis. The plant’s response to heat stress involves the heat-shock transcription factor A1 (HsfA1) that is indispensable in the activation of transcriptional networks. It is responsible for regulating the level of transcription factors expression, including the dehydration-responsive element binding protein 2A (DREB2A). However, the activity of HsfA1 is regulated by interaction with HSPs [26,27].

In this study, we explored the effect of heat stress on protein expression in two accessions of buckwheat, Panda and PA15. The total protein content increased under heat stress in both buckwheat accessions, but none of the proteins were newly expressed in response to heat stress. Up-regulation of protein expression may indicate a positive effect of high temperature on vegetative and reproductive development. However, the two studied accessions responded differently to heat stress, as illustrated by their different proteomes. In all organs, heat stress up-regulated more proteins in PA15 than in Panda. In our previous studies, the different responses of these accessions’ to heat stress were also reflected in the content of hormones [9] and HSP-70 and HSP-90 proteins [28] in their flowers and leaves.

Characteristic protein spots for the large Rubisco subunit were observed in the 2-D gels of proteins extracted from buds, open flowers, and wilted flowers. We also detected some chloroplast-localized proteins related to photosynthesis, because the floral tissues that the proteins were extracted from included the green pedicel. To identify proteins from gel spots we used nanoLC-MS/MS. It is the most adequate method used for this purpose, but may not be of use for low-abundant proteins [29]. In many cases, the analyzed sample had insufficient protein concentration; therefore, in the second attempt, the proteins were identified using the PMF technique. The PMF method requires less protein and is also much cheaper, but has many limitations that influenced the number of proteins identified in the experiment. The method fails to identify mixture proteins, low molecular mass proteins, and protein fragments. Additionally, PMF raises problems with the identification of large proteins. The success of the analysis is also determined by the presence of the protein sequences of interested search in the database [30]. In our experiment, among the proteins identified, none were specific to common buckwheat. All of them were homologous to proteins in different species. Similar results were presented by other authors when studying poorly known species [31]. In future studies on the buckwheat proteome in protein identification, a buckwheat genome database should be included [32]. This approach will allow to identify even proteins that we have not been able to identify so far. However, a potential change in the identification methods used would not change the overall picture obtained in our study and thus the conclusions drawn from our research on buckwheat response to heat stress. On the other hand, a change in methodology could be useful if the aim of our work was to identify candidate genes for high temperature stress tolerance.

Four proteins were up-regulated by heat stress in the flower buds of both accessions. These proteins included acyl-[acyl-carrier-protein] (ACP) desaturase 6, 60S ribosomal protein L5-1, and the trihelix transcription factor GTL2. ACP desaturase 6 is localized in the chloroplasts. It is responsible for unsaturated fatty acid biosynthesis, and its role is to introduce a double bond during esterification of the acyl group to the acyl carrier protein. Derivatives of unsaturated fatty acids (UFA) are known to function as signaling molecules in responses to various stresses. High temperature has been shown to increase the UFA content in olive plants [33]. Changes to the UFA content have been shown to affect the stress response, and result in changes in salicylic acid (SA)- and jasmonic acid (JA)-mediated defense responses, especially to biotic stresses [34]. In our previous study, we found that high temperature led to increased SA contents in buds of Panda and PA15, but decreased the contents of JA and its methyl ester (JA-Met) in both accessions [9]. The other two identified proteins induced by heat stress in the two accessions were related to protein synthesis pathways. The 60S ribosomal protein L5-1 is a component of the ribosome, whereas the trihelix transcription factor GTL2 binds to a specific DNA sequence to regulate gene transcription. Magwanga et al. [35] also found that the trihelix transcription factor GTL2 was highly up-regulated in cotton under drought and salt stress conditions.

In open flowers, only 6-phosphogluconate dehydrogenase (6PGDH) was up-regulated by heat stress in both buckwheat accessions. This enzyme plays a key role in the oxidative pentose phosphate pathway (OPPP), which is critical for maintaining redox balance under stress situations. 6PGDH probably controls the efficiency of this pathway. In rice, expression of the gene encoding 6PGDH was found to be up-regulated by abscisic acid (ABA) treatment [36]. In our previous study, we found that the free ABA content in open flowers increased in Panda and decreased in PA15 under heat stress. However, the concentration of the conjugate ABA-glc increased in PA15 but remained stable in Panda under heat stress [9].

Heat stress resulted in a dramatic increase in the abundance of one protein spot in the open flowers of Panda (to 213 times that in control plants grown at 20 °C). We identified this protein as the indole-3-glycerol phosphate synthase (IGPS) chloroplastic-like isoform X2. This enzyme produces indole-3-glycerol phosphate (IGP) as the precursor of indole ring-containing compounds and participates in the biosynthesis of tryptophan, indole 3-acetic acid, phytoalexin alkaloids, and glucosinolates. Indole plays roles in abiotic and biotic stress responses, but also in flowers, where it is emitted as a scent to attract pollinators [37]. IGP may be a branchpoint compound in the tryptophan-independent and tryptophan-dependent auxin biosynthetic pathways [38]. Auxins are responsible for plant fertility and high temperatures reduce plant fertility through repression of expression of the YUCCA auxin biosynthesis genes [39]. YUCCA (YUC)-type flavin-containing monooxygenases catalyze a reaction whose product is indole 3-acetic acid [40]. Thus, in this context, it can be speculated that the increase in IGPS accumulation in the flowers of the heat-sensitive Panda may be an attempt to counteract the reduction in auxin content in heat-treated flowers observed in this accession in contrast to the tolerant PA15, where this decrease was lower [9]. It is possible that one of the possible heat-tolerance mechanism in PA15 is the lower heat-sensitivity of some elements of auxin biosynthesis pathway.

Few of the proteins showing heat-induced changes in abundance in wilted flowers were identified, especially those in PA15. Many of the protein spots on the 2-D gels of proteins from wilted flowers were probably protein fragments resulting from an increase in proteolytic enzyme activity and limited repair mechanisms. Wilted flowers were aborted due to a lack of pollination. Proteins can be fragmented by reactive oxygen species (ROS) and proteolytic enzymes. Heat stress accelerates the generation and reactions of ROS, and senescing tissues do not have an efficient antioxidant system [41]. In wilted flowers, nine protein spots accumulated under heat stress in both accessions. One of them could not be identified. Two spots were identified as HSP-70s. Members of the HSP-70 family function as chaperones to facilitate protein folding, degradation, complex assembly, and translocation. They play a key role in stabilizing proteins under optimal and stress conditions [42]. Previously, we detected the accumulation of HSP-70 and HSP-90 in buckwheat flowers at various stages of development [28]. Moreover, we identified the chaperone protein ClpC in the HSP-100 family that plays a vital role in chloroplast function [43]. The presence of HSP-70 and HSP-100 proteins suggests that certain defense mechanisms function in wilted flowers, but they may be involved in the proper degeneration of the organ. Another protein expressed under heat stress in both Panda and PA15 was the V-type proton ATPase (V-ATPase) catalytic subunit A, which is a component of the membrane-bound V-ATPase located at the tonoplast and other sites in the endomembrane system of plant cells. The abundance of V-ATPase subunits is known to be modulated by environmental stresses [44]. Another heat-affected protein in wilted flowers was the calpain-type cysteine protease DEK1, the only calpain protein in plants. This protein is essential for embryo development [45]. The glutamate receptor 3.4 was also commonly up-regulated by heat stress in both accessions. This protein is a component of the glutamate receptor-like channel (GLR). GLRs play a role in calcium signaling during the response to environmental stresses [46]. Two proteins up-regulated by heat stress in wilted flowers were involved in carbohydrate metabolism: cytosolic phosphoglycerate kinase (PGK) and chloroplastic fructose-bisphosphate aldolase (FBA). Cytosolic PGK is involved in glycolysis and gluconeogenesis. Plants also have plastidial isoforms of PGK that may simultaneously participate in the Calvin–Benson cycle and glycolytic/gluconeogenic reactions [47]. Chloroplastic FBA is a bifunctional enzyme involved in the formation of fructose-1,6-bisphosphate (FBP) and sedoheptulose-1,7-bisphosphate (SuBP) in the Calvin cycle. It also functions as a sedoheptulose/fructose-bisphosphate aldolase (SFBA). FBA aldolase activity, but not SuBP activity, is important for glycolytic and gluconeogenetic reactions in the cytoplasm [48].

In donor leaves, only *S*-adenosylmethionine synthetase (SAMS) was up-regulated by heat stress in both buckwheat accessions. This enzyme synthesizes *S*-adenosylmethionine (SAM) from ATP and L-methionine. It is involved in the biosynthesis of ethylene, nicotiamine, and polyamines. It represents the major hub of the methionine metabolism and participates in plant responses to environmental stresses [49].

## 4. Materials and Methods

### 4.1. Plant Material and Growth Conditions

Seeds of common buckwheat (*Fagopyrum esculentum* Moench), the Polish cultivar Panda and the PA15 strain, were supplied by Małopolska Plant Breeding (Polanowice, Poland), and were produced at the plant production facility in Palikije. Panda is more sensitive to heat stress than PA15 is, as manifested by the degeneration of a large number of embryo sacs [9]. The plant growth conditions in the phytotron have been described in our previous papers [8,9]. Plants were cultivated in plastic pots of 10 dm^3^ capacity (six plants per pot) filled with a mixture of commercial soil substrate (pH = 5.8) and perlite (1:1, *v:v*) under a 16 h photoperiod and 300 µmol m^−2^ s^−1^ (High-Pressure Sodium, HPS lamps, SON-T+ AGRO, Philips, Brussels, Belgium) at a humidity of 50–60%. For the first 3 weeks, all plants were grown at 20 °C, and then half of them were transferred to a chamber at 30 °C (heat stress) with the same humidity and light conditions. When the plants were 2 months old, we collected flowers at three developmental phases (buds, open flowers, and wilted flowers) and donor leaves (fully developed young leaves closest to the flower cluster) from plants in the control and heat stress treatments. The experiment was repeated twice. The samples were immediately frozen in liquid nitrogen and then stored at −80 °C until subsequent analysis. Protein extraction and electrophoretic separation were performed from three aggregate replicates for each development phase of flowers and donor leaves, for both accessions in both temperature treatments.

### 4.2. Protein Extraction

Total proteins were extracted using a phenol-based procedure [50] (modified by Hajduch et al. [51]). Buckwheat tissues were pulverized into a fine powder in liquid nitrogen with a mortar and pestle. The powder was suspended in 10 cm^3^ of a phenol-based extraction buffer (50% [*v/v*] phenol, 0.45 M sucrose, 5 mM EDTA, 0.4% [*v/v*] 2-mercaptoethanol, 50 mM Tris-HCl pH 8.8). The homogenate was allowed to reach room temperature, transferred to a Falcon tube, and shaken for 30 min. The phenol and aqueous phases were separated by centrifugation (5000 *g*, 15 min, 4 °C). Proteins were precipitated with five volumes of ice-cold 0.1 M ammonium acetate in 100% methanol at −20 °C for 16 h. After centrifugation (5000× *g*, 10 min, 4 °C), the protein pellet was washed twice with the precipitation solution, then with 80% acetone, and then with 70% ethanol. The total protein extracts were dissolved in 200 µL isolectricfocusing (IEF) sample solution (8 M urea, 2 M thiourea, 4% (*w/v*) 3-[(3-Cholamidopropyl)dimethylammonio]-1-propanesulfonate (CHAPS), 50 mM dithiothreitol (DTT)) by shaking for 1 h. The protein concentration was determined using a 2D Quant Kit (GE Healthcare, Piscataway, NJ, USA). Protein extracts in the IEF sample solution were stored at −80 °C until analysis.

### 4.3. Two-Dimensional Gel Electrophoresis

Two-dimensional gel electrophoresis (2-DE) was based on the procedure recommended by GE Healthcare. The desired amount of protein (700 mg) was mixed with 4.6 µL IPG buffer (pH range 4–7) (GE Healthcare), adjusted to 450 µL with IEF sample solution, and loaded onto a 24 cm immobilized pH gradient strip (Immobiline DryStrip gel; GE Healthcare) with a linear pH range of 4–7. The strips were passively rehydrated in a DryStrip IPGbox (GE Healthcare) for 16 h. The first dimension of isoelectrofocusing (IEF) was carried out using an Amersham Ettan IPGphor II unit (GE Healthcare). The six-step focusing protocol with a current limit of 75 µA per strip was as follows: (a) 45 Vh at 150 V, (b) 375 Vh at 150 V, (c) 500 Vh at 500 V; (d) 800 Vh at 1000 V; (e) 16,500 Vh at 10,000 V; (f) 27,200 Vh at 10,000 V. After IEF, the strips were incubated in an equilibration buffer (1.5 M Tris-HCl pH 8.8, 6 M urea, 30% (*v/v*) glycerol, 5% (*w/v*) sodium dodecyl sulfate (SDS)) for 15 min with 2% (*w/v*) dithiothreitol (DTT) and then for another 15 min with 2.5% (*w/v*) iodoacetamide. After equilibration, each strip was placed onto a 12% SDS-polyacrylamide gel and then overlaid with 0.5% (*w/v*) agarose in an SDS running buffer with Coomassie Brilliant Blue G-250 as the tracking dye. Separation on the second dimension was performed using an SE900 Large Format Vertical Gel Protein Electrophoresis Unit (Hoefer Scientific Instruments, San Francisco, CA, USA) at 48 W per gel until the dye migrated off the gel. After electrophoresis, each gel was washed three times for 15 min each time in deionized water and then stained overnight in colloidal Coomassie staining solution (20% (*v/v*) ethanol, 1.6% (*v/v*) phosphoric acid, 8% (*w/v*) ammonium sulfate, 0.08% (*w/v*) Coomassie Brilliant Blue G-250) using the modified method of Neuhoff et al. [52].

### 4.4. Gel Image Analysis

Stained 2-DE gels were digitalized using an Epson Perfection V850 Pro scanner at a resolution of 300 dpi and 16-bit gray scale pixel depth. Gel images were analyzed using Delta2D software (Decodon, Greifswald, Germany). The volume of the spots was normalized against the total volume of all spots in the analysis. The *t* test value for each spot was calculated using Delta2D software. Only spots with a *p*-value lower than or equal to 0.05 were considered to be differentially expressed. Spots with at least 2.0-fold differences in protein abundance between two treatments were chosen for further analysis.

### 4.5. Protein Identification

The protein spots were manually selected and excised from the gels for identification. First, proteins were analyzed by liquid chromatography-mass spectrometry (nanoLC-MS/MS). If proteins could not be identified by nanoLC-MS/MS, peptide mass fingerprinting (PMF) analysis was performed. Mass spectrometry analysis was performed at the Laboratory of Proteomics and Mass Spectrometry, Maj Institute of Pharmacology, Polish Academy of Sciences, Kraków (Poland).

The excised protein spots were prepared for mass spectrometry analysis according to the protocol described by Hartman et al. [53]. The gel pieces in tubes were incubated at 40 °C in 100 mM ammonium bicarbonate for 10 min. Then, acetonitrile was added to a final concentration of 50% (*v/v*) and the mixture was incubated at 40 °C for 10 min. After incubation, the solution was removed. The washing step was repeated three times to remove all Coomassie dye, until the gels were completely colorless. Finally, the gels were dehydrated in anhydrous acetonitrile. The acetonitrile was removed, and then the dry gel pieces were reswollen in 25 µL 50 mM DTT dissolved in 100 mM ammonium bicarbonate, followed by incubation at 60 °C for 45 min. Then, the DTT solution was replaced with 25 µL 100 mM iodoacetamide in 100 mM ammonium bicarbonate. The gel pieces were incubated at room temperature in darkness for 20 min, then 25 µL 10 ng µL^−1^ trypsin Gold (Promega, Madison, WI, USA) solution in 100 mM ammonium bicarbonate was added and the gel pieces were incubated at 4 °C for 1 h. Then, 25 µL 50 mM ammonium bicarbonate solution was added and the gel pieces were incubated overnight at 37 °C. The next day, the supernatant containing the digested peptides was collected in a new tube and combined with subsequent fractions. The gel pieces were immersed in 50 mM ammonium bicarbonate and incubated at 40 °C for 10 min before adding acetonitrile to a final concentration of 50% (*v/v*). The resulting supernatants were collected. Further extraction of peptides was performed in acidic conditions by incubation with 5% (*v/v*) formic acid in 50% (*v/v*) acetonitrile, twice. The gel pieces were dehydrated in anhydrous acetonitrile. Combined solutions from one sample were dried and dissolved in 20 µL 0.1% formic acid and then analyzed by nanoLC-MS/MS and PMF.

The nanoLC-MS/MS analyses were performed using an Easy-nLC II nano capillary chromatography system (Bruker Daltonics, Bremen, Germany) as described in Drabik et al. [54]. Peptides were separated on a 3 µm Biosphere C18 column (10 cm long, 75 µm internal diameter; Nanoseparations, Nieuwkoop, The Netherlands). The gradient was formed using two mobile phases: Phase A: 0.1% formic acid in water; phase B: 0.1% formic acid in acetonitrile. The total flow rate was 300 nl min^−1^. The system was controlled using Hystar software (Bruker Daltonics). The gradient program was as follows: from 2% to 45% Phase B in 30 min, then 90% Phase B for 10 min, decreasing to 2% Phase B until 60 min for column equilibration. Fractions eluted from the column were directly deposited with a w matrix on the MALDI target plate by a Proteineerfc II sample collector (Bruker Daltonics). Fractions were collected at 15 s intervals. Samples made up of 96 fractions were spotted on a 384 MALDI target plate. α-Cyano-4-hydroxycinnamic acid was used as the MALDI matrix. The mass spectrometry analyses were performed on an Ultraflextreme instrument (Bruker Daltonics) in the positive ion mode.

Samples for PMF analysis were bound to C18 resin in ZipTip columns (Supel-Tips C18 PipetteTips, Supelco/Sigma-Aldrich, Bellefonte PA, USA) according to the manufacturer’s instructions. The peptides were eluted from the column with saturated α-cyano-4-hydroxycinnamic acid solution in 60% (*v/v*) acetonitrile with 0.1% (*v/v*) trifluoroacetic acid directly on the MALDI target plate.

The acquired mass spectra and fragment mass spectra (for both LC-MS and PMF) were analyzed using FlexAnalysis software (Version: 3.4, Bruker Daltonics) and ProteinScape (Version: 3.0.0 446, Bruker Daltonics) and were processed using the Mascot algorithm (Engine version: 2.3, Matrix Science) against the SwissProt_2015_04 database. The searches were performed with the following parameters: Cerbamidomethylation of cysteine as a fixed modification; oxidation of methionine as an allowable variable modification; up to 1 missed cleavage allowed; 25 ppm for precursor mass tolerance; 0.6 Da for MS/MS mass tolerance; peptide charge: 1+ for PMF (MALDI-TOF instrument-UltrafleXtreme from Bruker Daltonics) and 0.3 Da for precursor mass tolerance; 0.6 Da for MS/MS mass tolerance; peptide charge: 2+, 3+, 4+ for LC-MS (ESI-IT instrument on an Amazon SL from Bruker Daltonics) analyses. The database search was run against the protein database Viridiplantae (563,552 sequences; 203,007,781 residues; May 2020). If no protein was identified, the database was searched for all taxa. Proteins with a mascot score higher than 30 and with a level of false positives of *p* ≤ 0.05 were considered as identified. In cases where the identified protein belonged to an organism other than plants, a search was performed based on the amino acid sequence of the homologous protein among green plants using BLASTP (https://blast.ncbi.nlm.nih.gov).

## 5. Conclusions

Two common buckwheat accessions, Panda and PA15, differ in their tolerance to high temperature, as illustrated by the frequency of embryo sac degradation under heat stress. The different responses of the two accessions to heat stress were reflected in their protein profiles. All heat-affected proteins showed up-regulated expression in the organs of the two accessions. Many proteins could not be identified. It is possible that some protein spots were protein fragments resulting from proteolysis and inadequate repair mechanisms. There were more heat-affected proteins in PA15, the heat-tolerant accession, than in Panda, the heat-sensitive accession. Surprisingly, only a few proteins were commonly up-regulated by heat in both accessions. Plants’ tolerance to heat stress, as to other environmental stresses, is the sum of minor and major changes in the proteome, and cannot be explained by single changes. Proteins common to both accessions characterize the heat-affected protein profile of common buckwheat. The abundance of indole-3-IGPS chloroplastic-like isoform X2 increased markedly in open flowers of Panda under heat stress. This may be a candidate protein to serve as a marker of sensitivity of buckwheat plants to heat stress.

## Figures and Tables

**Table 1:** Results of protein identification performed on spots showing at least 2 times higher (*p* ≤ 0.005) abundance in plants grown at high temperature than in plants grown at control temperature. LC-MS/MS, liquid chromatography-mass spectrometry; PMF, mass fingerprinting.

Spot No. ^a^	Organ ^b^	Technique ^c^	UniProt No. ^d^	Protein Name ^e^	Reference Organism ^f^	M_t_ [kDa] ^g^	pI_t_^h^	Protein Score ^i^	Peptide Count ^j^	Coverage (%) ^k^
2	B	LC-MS/MS	RS122_ARATH	40S ribosomal protein S12-2	*Arabidopsis thaliana*	15.3	5.55	169.24	2	12.5
4	B	PMF	RL26_BRACM	60S ribosomal protein L26	*Brassica campestris*	16.9	11.60	135.90	4	8.9
5	B	PMF	EIF3A_MAIZE	Eukaryotic translation initiation factor 3 subunit A	*Zea mays*	111.5	9.80	134.80	4	5.8
6	B	PMF	CLDS_TOBAC	Copal-8-ol diphosphatehydratase, chloroplastic	*Nicotiana tabacum*	93.2	5.50	128.00	2	3.6
7	B	LC-MS/MS	PDRP2_ARATH	Pyruvate, phosphatedikinase regulatory protein 2	*Arabidopsis thaliana*	41.4	9.64	97.92	1	2.1
8	B	PMF	STAD6_ORYSI	Acyl-[acyl-carrier-protein] desaturase 6, chloroplastic	*Oryza sativa subsp. indica*	46.5	7.2	136.80	2	9.0
9	B	LC-MS/MS	PS4_PINST	Putative LRR diseaseresistance protein/transmembrane receptor kinase PS4 (fragment)	*Pinus strobus*	0.90	11.10	95.06	1	100.0
10	B	LC-MS/MS	AB5F_ARATH	ABC transporter F family member 5	*Arabidopsis thaliana*	78	6.49	94.56	1	1.7
13	B	PMF	RHM2_ARATH	Trifunctional UDP-glucose 4,6-dehydratase/UDP-4-keto-6-deoxy-D-glucose 3,5-epimerase/UDP-4-keto-L-rhamnose reductase RHM2	*Arabidopsis thaliana*	75.2	6.00	116.70	1	2.4
15	B	LC-MS/MS	PSA5_ORYSJ	Proteasome subunit alpha type-5	*Oryza sativa subsp. japonica*	26	4.60	557.20	11	37.1
16	B; OF	PMF	UPL1_ARATH	E3 ubiquitin-protein ligase UPL1	*Arabidopsis thaliana*	404.7	4.80	126.20	3	1.3
17	B	LC-MS/MS	PSMD4_ARATH	26S proteasome non-ATPase regulatory subunit 4 homolog	*Arabidopsis thaliana*	40.7	4.30	183.00	2	4.4
18	B; WF	PMF	Y1765_ARATH	Probable LRR receptor-likeserine/threonine-protein kinase At1g07650	*Arabidopsis thaliana*	112.8	9.50	118.90	3	4.0
20	B; OF	PMF	KN12D_ARATH	Kinesin-like protein KIN-12D	*Arabidopsis thaliana*	314.9	5.10	118.00	3	1.3
21	B; OF	LC-MS/MS	6PGD1_SPIOL	6-phosphogluconate dehydrogenase, decarboxylating 1	*Spinacia oleracea*	53.2	6.00	786.00	13	20.1
22	B	PMF	RL51_ARATH	60S ribosomal protein L5-1	*Arabidopsis thaliana*	34.3	9.70	130.40	3	11.6
23	B	PMF	QWRF4_ARATH	QWRF motif-containing protein 4	*Arabidopsis thaliana*	66.9	10.20	123.00	3	4.2
24	B	PMF	GTL2_ARATH	Trihelix transcription factor GTL2	*Arabidopsis thaliana*	71.2	6.70	113.50	2	2.7
25	B	PMF	KN12F_ORYSJ	Kinesin-like protein KIN-12F	*Oryza sativa subsp. japonica*	317.1	5.00	137.70	5	2.0
26	B	PMF	MYB98_ARATH	Transcription factor MYB98	*Arabidopsis thaliana*	50.1	6.10	129.70	4	6.8
27	B	LC-MS/MS	TPIC_ARATH	Triosephosphate isomerase, chloroplastic	*Arabidopsis thaliana*	33.3	8.90	355.70	5	16.5
28	B	LC-MS/MS	IPYR2_ARATH	Soluble inorganic pyrophosphatase 2	*Arabidopsis thaliana*	24.7	5.70	125.80	1	5.5
33	B; WF	LC-MS/MS	ADF2_ORYSJ	Actin-depolymerizing factor 2	*Oryza sativa subsp. japonica*	16.8	5.60	102.50	1	8.3
48	OF	LC-MS/MS	AB2C_ARATH	ABC transporter C family member 2	*Arabidopsis thaliana*	182	6.00	103.88	0	0.0
49	OF	PMF	HXK4_ORYSJ	Hexokinase-4, chloroplastic	*Oryza sativa subsp. japonica*	54.7	6.50	122.80	3	6.5
51	OF	PMF	SWTIE_ARATH	Protein SWEETIE	*Arabidopsis thaliana*	244.2	5.10	123.00	3	1.8
52	OF	PMF	CALS2_ARATH	Callosesynthase 2	*Arabidopsis thaliana*	225.9	9.20	132.50	4	1.9
53	OF	LC-MS/MS	PS4_PINST	Putative LRR diseaseresistance protein/transmembrane receptor kinase PS4 (fragment)	*Pinus strobus*	0.90	11.10	90.53	1	100.0
54	OF	LC-MS/MS	ZDHC8_ARATH	Probable protein S-acyltransferase 20	*Arabidopsis thaliana*	76.8	9.60	123.05	2	2.4
57	OF; WF	PMF	MDHC2_ARATH	Malate dehydrogenase 2, cytoplasmic	*Arabidopsis thaliana*	35.7	6.30	466.10	8	26.8
58	OF	PMF	UGDH2_ARATH	UDP-glucose 6-dehydrogenase 2	*Arabidopsis thaliana*	53.1	5.60	162.80	3	7.7
60	OF; WF	LC-MS/MS	HSP70_MAIZE	Heatshock 70 kDa protein	*Zea mays*	70.5	5.10	470.40	8	14.7
61	OF	PMF	AUG8_ARATH	AUGMIN subunit 8	*Arabidopsis thaliana*	69.8	10.70	117.50	4	5.7
77	WF	PMF	RLT2_ARATH	Homeobox-DDT domain protein RLT2	*Arabidopsis thaliana*	190.3	5.30	116.30	4	2.2
78	WF	PMF	RCA_MALDO	Ribulose bisphosphate carboxylase/oxygenase activase, chloroplastic	*Malus domestica*	48	8.20	277.70	5	13.5
79	WF	PMF	HSP7N_ARATH	Heatshock 70 kDa protein 18	*Arabidopsis thaliana*	68.3	5.20	539.30	7	16.4
80	WF	PMF	CLPC_PEA	Chaperone protein ClpC, chloroplastic	*Pisum sativum*	102.6	6.50	853.70	16	16.3
81	WF	PMF	VATA_BRANA	V-type proton ATPase catalytic subunit A	*Brassica napus*	68.7	5.10	356.60	6	9.0
82	WF	PMF	DEK1_ARATH	Calpain-typecysteineprotease DEK1	*Arabidopsis thaliana*	238.1	6.10	131.20	4	2.4
83	WF	PMF	GLR34_ARATH	Glutamate receptor 3.4	*Arabidopsis thaliana*	107.1	9.10	120.00	4	5.0
84	WF	PMF	C76AD_BETVU	Cytochrome P450 76AD1	*Beta vulgaris*	56.2	8.10	133.30	3	4.6
85	WF	PMF	ILV5_ARATH	Ketol-acid reductoisomerase, chloroplastic	*Arabidopsis thaliana*	63.8	6.40	181.00	2	4.9
86	WF	PMF	Y5537_ARATH	G-typelectin S-receptor-likeserine/threonine-protein kinase At5g35370	*Arabidopsis thaliana*	96	6.60	127.20	3	4.5
87	WF	LC-MS/MS	PGKY_TOBAC	Phosphoglycerate kinase, cytosolic	*Nicotiana tabacum*	42.3	5.60	557.90	10	28.9
88	WF	PMF	ALFP2_ARATH	Fructose-bisphosphatealdolase 2, chloroplastic	*Arabidopsis thaliana*	43	6.80	451.50	8	15.8
89	WF	PMF	SMC3_ARATH	Structural maintenance of chromosomes protein 3	*Arabidopsis thaliana*	139.3	6.10	124.50	4	2.9
91	WF	LC-MS/MS	1433_HELAN	14-3-3-like protein	*Helianthus annuus*	28.9	4.50	356.30	6	19.7
92	WF	LC-MS/MS	HSP7C_PETHY	Heatshock cognate 70 kDa protein	*Petunia hybrida*	71.2	5.00	1145.10	20	30.7
93	WF	LC-MS/MS	SPD1_DATST	Spermidine synthase 1	*Datura stramonium*	34	5.10	246.70	3	8.8
95	WF	LC-MS/MS	GDI_ARATH	Guanosine nucleotide diphosphate dissociation inhibitor	*Arabidopsis thaliana*	49.5	5.00	266.90	5	10.1
96	WF	LC-MS/MS	PMG2_ARATH	Probable 2,3-bisphosphoglycerate-independent phosphoglyceratemutase 2	*Arabidopsis thaliana*	60.7	5.50	267.00	3	6.8
97	WF	LC-MS/MS	PMGI_MESCR	2,3-bisphosphoglycerate-independent phosphoglycerate mutase	*Mesembryanthemum crystallinum*	61.1	5.30	340.60	6	12.7
98	WF	LC-MS/MS	RH15_ARATH	DEAD-box ATP-dependent RNA helicase 15	*Arabidopsis thaliana*	48.3	5.30	369.40	6	12.6
99	WF	LC-MS/MS	ALF_ORYSJ	Fructose-bisphosphate aldolase cytoplasmic isozyme	*Oryza sativa subsp. japonica*	38.8	7.70	254.80	4	7.8
100	WF	LC-MS/MS	GLYG1_SOYBN	Glycinin G1	*Glycine max*	55.7	5.80	394.60	8	21.0
101	WF	LC-MS/MS	UPTG_MAIZE	Alpha-1,4-glucan-protein synthase [UDP-forming]	*Zea mays*	41.2	5.70	423.00	9	27.7
102	WF	LC-MS/MS	PSA1_ORYSJ	Proteasome subunit alpha type-1	*Oryza sativa subsp. japonica*	29.6	5.60	151.70	2	10.0
153	L	LC-MS/MS	CAHC_TOBAC	Carbonic anhydrase, chloroplastic	*Nicotiana tabacum*	34.5	6.46	139.33	1	3.1
154	L	LC-MS/MS	CAHC_TOBAC	Carbonic anhydrase, chloroplastic	*Nicotiana tabacum*	34.5	6.46	110.55	1	5.6
158	L	PMF	METK_CAMSI	S-adenosylmethionine synthase	*Camellia sinensis*	42.8	5.20	150.70	3	6.9
160	L	PMF	PP207_ARATH	Pentatricopeptide repeat-containing protein At3g02330, mitochondrial	*Arabidopsis thaliana*	101.6	6.00	128.60	3	4.9
161	L	PMF	CHR4_ARATH	Protein CHROMATIN REMODELING 4	*Arabidopsis thaliana*	247.8	5.90	128.70	4	3.2
162	L	LC-MS/MS	CAHC_PEA	Carbonic anhydrase, chloroplastic	*Pisum sativum*	35.4	7.74	263.63	1	5.5
163	L	LC-MS/MS	CDSP_ARATH	Thioredoxin-like protein CDSP32, chloroplastic	*Arabidopsis thaliana*	33.7	9.40	214.50	3	7.9
164	L	LC-MS/MS	CYSKP_SPIOL	Cysteine synthase, chloroplastic/chromoplastic	*Spinacia oleracea*	40.6	7.60	211.70	4	12.5
165	L	PMF	CRK20_ARATH	Putativecysteine-rich receptor-like protein kinase 20	*Arabidopsis thaliana*	74	6.60	118.50	3	7.1
166	L	PMF	RUB2_BRANA	RuBisCO large subunit-binding protein subunit alpha, chloroplastic	*Brassica napus*	61.6	5.00	569.20	9	15.8
167	L	PMF	TKTC_SPIOL	Transketolase, chloroplastic	*Spinacia oleracea*	80.2	6.20	262.90	5	6.5
168	L	PMF	HUAL1_ARATH	Protein HUA2-LIKE 1	*Arabidopsis thaliana*	156.5	8.90	113.20	3	2.4
169	L	PMF	GRDP1_ARATH	Glycine-richdomain-containing protein 1	*Arabidopsis thaliana*	89.4	6.60	129.60	5	6.7
170	L	PMF	FENR1_ORYSI	Ferredoxin--NADP reductase, leaf isozyme 1, chloroplastic	*Oryza sativa subsp. indica*	40	8.70	192.90	3	7.7
171	L	PMF	KN12F_ORYSJ	Kinesin-like protein KIN-12F	*Oryza sativa subsp. japonica*	317.1	5.00	123.70	4	1.9
172	L	PMF	GLTB2_ARATH	Ferredoxin-dependent glutamatesynthase 2, chloroplastic	*Arabidopsis thaliana*	177.6	6.60	128.00	4	2.8

^a^ Spot number in 2-D gels; ^b^ buckwheat organs containing identified proteins; B—buds, OF—open flowers, WF—wilted flowers, L—leaves; ^c^ technique used to identify protein; ^d^ Uniprot reference number of protein; ^e^ homologous protein name from the UniProt/NCBI database; ^f^ organism from which protein is derived; ^g^ Mt—theoretical mass weight obtained from protein database; ^h^ pI_t_—theoretical isoelectric point obtained from protein database; ^i^ statistical probability of true positive identification of predicted protein; ^j^ amino acid sequence coverage of identified protein; ^k^ percentage of sequence covered by matched peptides.

**Table 2:** Corresponding plant homologs of non-plant proteins identified in nanoLC-MS/MS analysis. Homologous proteins were found using the protein–protein program (BLASTP) at NCBInr.

Spot No. ^a^	Organ ^b^	UniProt No.	Protein Name ^c^	Reference Organism ^d^	M_t_^e^ [kDa]	pI_t_^f^	Protein Score ^g^	Peptide Count ^h^	Coverage [%] ^i^	Homologous Protein Name ^j^	NCBI No.	Reference Organism ^f^	I ^k^	P ^l^
3	B	CAPZA_KLULA	F-actin-capping protein subunit alpha	*Kluyveromyces lactis*	29.9	4.54	99.31	1	4.2	F-actin-capping protein subunit alpha-like	XP_023907009.1	*Quercus suber*	33%	53%
11	B; WF	MTNC_GLUDA	Enolase-phosphatase E1	*Gluconacetobacter diazotrophicus*	24.9	4.97	97.57	1	3.0	Probable bifunctional methylthioribulose-1-phosphate dehydratase/enolase-phosphatase E1 1	XP_028794642.1	*Prosopis alba*	39%	53%
19	B	IF2_THEFY	Translation initiation factor IF-2	*Thermobifida fusca*	100.5	9.82	101.74	1	1.2	Translation initiation factor IF-2, chloroplastic	GEZ89434.1	*Tanacetum cinerariifolium*	52%	71%
50	OF	MURA_PSEU5	UDP-N-acetylglucosamine 1-carboxy-vinyltransferase	*Pseudomonas stutzer*	44.6	5.62	94.32	1	2.1	Glutamate synthase 1 [NADH], chloroplastic isoform X1	GEU28314.1	*Tanacetum cinerariifolium*	59%	74%
56	OF	LEXA_MYXXD	LexA repressor	*Myxococcus xanthus*	24.7	9.29	99.27	1	5.4	DNA-3-methyladenine glycosylase 1	GEX09398.1	*Tanacetum cinerariifolium*	37%	58%
59	OF	TRPC_ACIF2	Indole-3-glycerol phosphate synthase	*Acidithiobacillus ferrooxidans*	28.7	5.02	101.36	1	3.4	Indole-3-glycerol phosphate synthase, chloroplastic-like isoform X2	XP_026448585.1	*Physcomitrella patens*	48%	63%
159	L	SCP_CHIOP	Sarcoplasmic calcium-binding protein (fragment)	*Chionoecetes opilio*	0.8	11.1	114.44	1	100.0	F-box protein At3g58530 isoform X1	XP_021283280.1	*Herrania umbratica*	87%	100%

^a^ Spot number in 2-D gels; ^b^ buckwheat organs containing identified proteins; B—buds, OF—open flowers, WF—wilted flowers, L—leaves; ^c^ protein name from the UniProt database; ^d^ organism from which protein is derived; ^e^ Mt—theoretical mass weight obtained from protein database; ^f^ pI_t_—theoretical isoelectric point obtained from protein database; ^g^ statistical probability of true positive identification of the predicted protein; ^h^ amino acid sequence coverage of identified protein; ^i^ percentage of sequence covered by matched peptides; ^j^ homologous protein name from the NCBI database; ^k^ identity—extent to which two amino acid sequences match; ^l^ positives—similarities based on scoring matrix.

## Data Availability

The data supporting the findings of this study are available from the corresponding author upon reasonable request.

## Supplementary Materials

The following are available online at https://www.mdpi.com/1422-0067/22/5/2678/s1, Table S1: List of heat-affected proteins in buds, open flowers, wilted flowers, and donor leaves of common buckwheat accessions Panda and PA15; comparison of protein abundance between heat-treated plants and those growing in optimal conditions.

## Abbreviations

2-DE	Two-dimensional gel electrophoresis
6PGDH	6-phosphogluconate dehydrogenase
ABA	Abscisic acid
ACP	Acyl-[acyl-carrier-protein]
CHAPS	3-[(3-cholamidopropyl)dimethyl-ammonio]-1-propane sulfonate
DREB2A	Dehydration-responsive element binding protein 2A
DTT	Dithiothreitol
FBA	Fructose-bisphosphate aldolase
FBP	Fructose-1,6-bisphosphate
GLR	Glutamate receptor-like channel
HSF	Heat-shock transcription factor
HsfA1	Heat-shock transcription factor A1
HSP	Heat-shock proteins
IEF	Isoelectricfocusing
IGP	Indole-3-glycerol phosphate
IGPS	Indole-3-glycerol phosphate synthase
JA	Jasmonic acid
JA-Met	Methyl ester of jasmonic acid
OPPP	Oxidative pentose phosphate pathway
PGK	Phosphoglycerate kinase
PMF	Peptide mass fingerprinting
ROS	Reactive oxygen species
SA	Salicylic acid
SAM	*S*-adenosylmethionine
SAMS	*S*-adenosylmethionine synthetase
SDS	Sodium dodecyl sulfate
SFBA	Sedoheptulose/fructose-bisphosphate aldolase
SuBP	Sedoheptulose-1,7-bisphosphate
TF	Transcription factor
UFA	Unsaturated fatty acids
V-ATPase	V-type proton ATPase
